# The Role of the Left Anterior Temporal Lobe for Unpredictable and Complex Mappings in Word Reading

**DOI:** 10.3389/fpsyg.2017.00517

**Published:** 2017-04-05

**Authors:** Marilyne Joyal, Simona M. Brambati, Robert J. Laforce, Maxime Montembeault, Mariem Boukadi, Isabelle Rouleau, Joël Macoir, Sven Joubert, Shirley Fecteau, Maximiliano A. Wilson

**Affiliations:** ^1^Centre de Recherche de l’Institut Universitaire en Santé Mentale de Québec and Département de Réadaptation, Université Laval, Québec CityQC, Canada; ^2^Centre Interdisciplinaire de Recherche en Réadaptation et Intégration Sociale and Département de Réadaptation, Université Laval, Québec CityQC, Canada; ^3^Centre de Recherche de l’Institut Universitaire de Gériatrie and Département de Psychologie, Université de Montréal, MontréalQC, Canada; ^4^Clinique Interdisciplinaire de Mémoire, Centre Hospitalier Universitaire de Québec and Département des Sciences Neurologiques, Université Laval, Québec CityQC, Canada; ^5^Centre de Recherche du Centre Hospitalier de l’Université de Montréal, Axe Neurosciences et Département de Psychologie, Université du Québec à Montréal, MontréalQC, Canada

**Keywords:** anterior temporal lobe (ATL), reading, exception words, regular words, semantic variant of primary progressive aphasia (svPPA), Alzheimer’s disease (AD)

## Abstract

The anterior temporal lobes (ATLs) have been consistently associated with semantic processing which, in turn, has a key role in reading aloud single words. This study aimed to investigate (1) the reading abilities in patients with the semantic variant of primary progressive aphasia (svPPA), and (2) the relationship between gray matter (GM) volume of the left ATL and word reading performance using voxel-based morphometry (VBM). Three groups of participants (svPPA, Alzheimer’s Disease, AD and healthy elderly adults) performed a reading task with exception words, regular words and pseudowords, along with a structural magnetic resonance imaging scan. For exception words, the svPPA group had a lower accuracy and a greater number of regularization errors as compared to the control groups of healthy participants and AD patients. Similarly, for regular words, svPPA patients had a lower accuracy in comparison with AD patients, and a greater number of errors related to complex orthography-to-phonology mappings (OPM) in comparison to both control groups. VBM analyses revealed that GM volume of the left ATL was associated with the number of regularization errors. Also, GM volume of the left lateral ATL was associated with the number of errors with complex OPM during regular word reading. Our results suggest that the left ATL might play a role in the reading of exception words, in accordance with its role in semantic processing. Results further support the involvement of the left lateral ATL in combinatorial processes, including the integration of semantic and phonological information, for both exception and regular words.

## Introduction

In the last decade, the anterior temporal lobes (ATLs) have been recognized to play a key role in semantic processing (Patterson et al., 2007; Price, 2010; Visser et al., 2010). Specifically, the ventral ATLs, which include portions of the anterior fusiform and inferior temporal gyri, have been linked to processing various types of stimuli such as pictures, environmental sounds, words (Visser and Lambon Ralph, 2011; Visser et al., 2012; Chen et al., 2016) and faces (Collins et al., 2016; De Winter et al., 2016; Harry et al., 2016; Jonas et al., 2016). Therefore, the ventral ATLs appear to be involved in the conceptual processing of stimuli, thus playing a central role in semantic processing (Binney et al., 2010; Collins et al., 2016). The lateral ATLs, corresponding to the anterior middle temporal gyri, have also been associated with multimodal semantic processing (Visser et al., 2012). For instance, low-frequency repetitive transcranial magnetic stimulation (rTMS) delivered to the left lateral ATL (Pobric et al., 2007; Jackson et al., 2015) or both lateral ATLs (Lambon Ralph et al., 2009; Pobric et al., 2009) resulted in slower reaction times in synonym judgment tasks. rTMS applied to the lateral ATLs also slowed reaction times when participants had to perform an associative-semantic task with pictures (Pobric et al., 2010) or a picture naming task (Pobric et al., 2007). Therefore, the lateral ATLs may be involved in processing of meaningful concepts, either presented in written or pictorial forms.

Written stimuli can be read aloud in two different ways: using sublexical reading or whole-word reading (Cattinelli et al., 2013; Taylor et al., 2013; Danelli et al., 2015). Normally, whole-word reading implies semantic processing, allowing access to the meaning of words. However, theoretical models of reading make different predictions with regards to the role of semantics in whole-word reading. According to the Dual Route Cascaded model (DRC; Coltheart et al., 2001), all known words can be read through the lexical route without necessarily accessing semantic information. The DRC model therefore suggests that there is no causal relationship between the degradation of semantics and exception word reading impairment. When both deficits co-occur, they would arise from concurrent lesions to functionally unrelated regions (Coltheart et al., 2010). On the contrary, the connectionist triangle model of reading aloud (PDP; Seidenberg and McClelland, 1989; Plaut et al., 1996; Harm and Seidenberg, 2004) states that reading involves the computation of orthographic, phonological and semantic information. This model proposes two pathways of visual word processing: a phonological pathway (O→P) and a semantic pathway (O→S→P). With reading experience, the phonological pathway would become more involved in reading of consistent orthography-to-phonology mappings (OPM), and the semantic pathway would contribute significantly to the pronunciation of low-frequency exception words (Plaut et al., 1996), that do not follow the OPM of a given language (e.g., *yacht* and *colonel*). Thus, the connectionist triangle model of reading predicts that an impairment of the semantic pathway would result in impaired reading of exception words (Plaut et al., 1996).

On the other hand, sublexical reading involves the computation of each sub-unit (i.e., graphemes into their corresponding phonemes) of words. These subunits are processed according to regular OPM. New words and pseudowords are read through sublexical reading. Pseudowords are invented pronounceable letter sequences that have no semantic representations and thus need to be read on the basis of OPM (Brambati et al., 2009; Wilson et al., 2012; Chapleau et al., 2015). Thus, according to the PDP but not the DRC models, exception word reading relies more on semantic processing than does pseudoword reading. Regular words, which have both semantic representations and follow OPM, can be read via whole-word reading or sublexical reading.

Scripts differ across languages according to the transparency (or opacity) of their OPM. In that regard, cross-linguistic differences in the transparency/opacity continuum of a script can be associated with two separate concepts: predictability and complexity (Schmalz et al., 2015). Predictability refers to the degree to which words can be read on the basis of regular OPM. Thus, regular words are highly predictable, while exception words are unpredictable. Reading exception words by reliance on typical OPM would result in regularization errors (for instance, reading *pint* to rhyme with *mint*). The high prevalence of this type of regularization error for exception words is observed both in healthy normal adult readers (Chapleau et al., 2015) and in neurological patients such as post-stroke aphasia (Binder et al., 2016). Regularization errors are also a key feature in the reading profile of patients with the semantic variant of primary progressive aphasia (svPPA), who have both semantic (Rogers and Friedman, 2008) and reading impairments (Wilson et al., 2012; Woollams et al., 2014). Specifically, svPPA patients typically show surface dyslexia, a selective impairment of exception word reading that affects mostly low-frequency words (Wilson et al., 2012; Woollams et al., 2014; Boukadi et al., 2016). This shows that whole-word reading is impaired in such patients.

The other construct of orthography-to-phonology transparency/opacity relates to the complexity of OPM (Schmalz et al., 2015). OPM complexity has an influence on reading performance. For instance, a higher error rate has been observed in the reading of low-frequency Italian words with complex OPM as compared to words with simple OPM (Burani et al., 2006). Similarly, complex OPM affect reading performance in Dutch (Bosman et al., 2006) as well as reading acquisition in English and Italian (Marinelli et al., 2016). In French, complex mappings have been shown to affect spelling in brain damaged patients, who showed a specific deficit in the writing of stimuli with complex mappings (Auclair-Ouellet et al., 2013). However, to our knowledge, complex OPM have not been studied in reading in French. As compared to English, French is highly predictable as it has a higher percentage of words with regular OPM, but an extremely high number of such OPM are complex (Schmalz et al., 2015). For example, in French, context-sensitive OPM (e.g., the letter ‘g’ is pronounced /g/ in front of the vowels ‘a, o, u’ and /ʒ/ in front of the letters ‘e, i, y’) or OPM involving multi-letter graphemes (e.g., ‘eau’ is pronounced /o/) are complex (Schmalz et al., 2015). Errors with complex OPM can thus occur in both regular words and pseudowords. However, when it comes to regular word reading, it has been argued that semantics helps to compute phonology, especially when decoding is difficult (Boukrina et al., 2015). This suggests that semantics may support complex OPM reading in regular words. Thus, given the specificities of the French script in terms of a high number of complex OPM, analyzing this type of error would highlight the contribution of complexity to French orthographic opacity.

Two distinct neural networks have been associated with whole-word and sublexical reading. Price (2012) conducted a review of neuroimaging studies published between 1992 and 2011 and concluded that sublexical reading relies more on a pathway connecting the superior temporal gyrus with the ventral portions of the inferior parietal cortices and the dorsal precentral gyrus. On the other hand, whole-word reading relies on a pathway linking the left ventral occipito-temporal cortex and the left ventral inferior frontal gyrus. Taylor et al. (2013) conducted a meta-analysis based on 36 neuroimaging studies that contrasted patterns of activation in response to different word types (exception, regular and pseudowords). Their study provided additional details regarding the contribution of the ventral temporal cortex. They found that the left anterior fusiform gyrus, a region corresponding to the ventral ATL, was activated during word reading as compared to pseudoword reading. The left posterior fusiform gyrus and the occipitotemporal cortex were rather activated during pseudoword reading, as compared to word reading. Their results suggest that the ventral ATL plays a role in lexical/semantic processing of word reading. This finding has been replicated by Hoffman et al. (2015) in a recent functional magnetic resonance imaging (fMRI) study. Specifically, healthy participants performed a reading task with regular and exception words of high and low frequency inside the scanner. Reading aloud both regular and exception low-frequency words elicited greater activation of the left ventral ATL as compared to high-frequency words. Low-frequency words pose greater processing demands and would thus benefit more from semantic support, which provides additional input to that of the phonological pathway. This could lead to greater activation in regions involved in semantic processing for low-frequency words as compared to high-frequency words (Taylor et al., 2013). In another study, Shimotake et al. (2015) applied high-frequency electrical cortical stimulation to the left or right ventral ATL with subdural electrodes in patients with epilepsy or brain tumor. During stimulation of the left ventral ATL, kanji word reading (i.e., ideograms with semi-opaque orthography) was consistently impaired, while kana word reading (i.e., a syllabic script with transparent OPM) was relatively spared. However, whether these results can be generalized to alphabetical codes remains unclear. The fact that Taylor et al. (2013) did not find greater activation of the ventral ATL during exception word reading as compared to regular word reading in alphabetic scripts such as English or French seems to challenge this idea.

Regarding the lateral ATLs, Taylor et al. (2013) did not find any differential activation in these regions for words as compared to pseudowords, or for exception as compared to regular words. One possible explanation is that fMRI is sensitive to magnetic field inhomogeneities (Visser et al., 2010). Varying magnetic susceptibilities of bone, brain and air in the area of the ATLs can particularly lead to signal dropout and distortion. Consequently, the activation of the ATLs is less likely to be observed. Nonetheless, distorsion-corrected fMRI can overcome this susceptibility artifact. In an fMRI study, Wilson et al. (2012) acquired EPI images in an axial plane aligned with the hippocampus and reduced slice thickness to minimize signal distortion in the ATLs. They found significant activation of the left anterior middle temporal gyrus during exception word reading in comparison to pseudoword reading. Also, using distortion-correction, Hoffman et al. (2015) recently found that the left lateral ATL was more activated during exception word reading as compared to regular word reading. This was the case only for individuals who relied to a larger extent on semantic knowledge while reading aloud. In their study, the involvement of semantics in reading was measured with the size of the consistency effect, calculated from the performance difference between low-imageability regular and exception words. Considering that low-imageability words have weak semantic representations (Plaut and Shallice, 1993), individuals who rely more on semantic knowledge when reading aloud have greater difficulties to read these words and especially exception words. On the contrary, individuals with an efficient direct pathway between orthographic and phonological information require little semantic support to read exception words, and show small consistency effects (Hoffman et al., 2015). However, whether there is an involvement of both ventral and lateral ATLs in reading of exception and regular words in alphabetical scripts remains elusive. Analyzing the number of errors with both unpredictable and complex OPM, in exception and regular words, respectively, may shed light on the involvement of the left ATL in whole-word reading.

Thus, the goals of the present article are: (1) to characterize the reading abilities in individuals with svPPA, known to have reading difficulties related to whole-words, and to compare their performance with two control groups, one of healthy participants and another of patients suffering from Alzheimer’s disease (AD); and (2) to investigate whether gray matter (GM) volume of the ATLs correlates with the number of regularization errors and errors with complex OPM, for exception and regular word reading, respectively, using voxel-based morphometry (VBM). The primary reason why we conducted VBM analyses is the limited available data regarding structural brain-behavior correlations with svPPA patients. For a meta-analysis of studies on the pattern of atrophy in the svPPA, see Chapleau et al. (2016). Behaviorally, we hypothesize that, as compared to the healthy participants, the svPPA group will have a lower reading accuracy for exception words and a greater number of regularization errors (Wilson et al., 2012; Woollams et al., 2014; Boukadi et al., 2016). We also hypothesize that the svPPA group will have a greater number of errors with complex OPM in regular word reading. This hypothesis is based on the assumptions that svPPA patients have semantic impairment (Rogers and Friedman, 2008) and that semantics supports difficult phonological decoding (Boukrina et al., 2015). As compared to AD patients, svPPA will also show a lower reading accuracy for exception words and a greater number of regularization errors. Since the AD patients from this study are matched to the svPPA patients in terms of age and education, they are at a minimal stage of disease progression. Then, as in Patterson et al. (1994), we expect AD patients to exhibit a similar reading pattern as that of healthy controls in terms of accuracy. Indeed, Patterson et al. (1994) formed three subgroups of AD patients (minimal, mild, and moderate) based on their score on the Mini-Mental State Examination (Folstein et al., 1975). Only mild and moderate but not minimal groups had lower number of correct responses in a reading task as compared to healthy controls. The average MMSE score of our AD group (25 ± 2.7) is comparable to that of their minimal group (25.6 ± 1.9), which had intact reading accuracy in their study.

From an anatomical point of view, based on Taylor et al. (2013) and Hoffman et al. (2015) findings, we postulate that the volume of the left ATL will be associated with the number of regularization errors, since this type of error is associated with exception word reading. We also hypothesize that the volume of the left ATL will be associated with the number of errors with complex OPM during regular word reading, as semantics can help computation of phonology under this seemingly difficult decoding (Boukrina et al., 2015) and as the ATLs are involved in semantic processing (Price, 2010; Visser and Lambon Ralph, 2011; Rice et al., 2015).

## Materials and Methods

### Participants

Nine svPPA patients, 11 healthy control participants, and 12 AD control patients participated in the study. The three groups were matched on age, education and sex (see **Table 1** for demographic information and neuropsychological data). Patients were recruited through the interdisciplinary memory clinic of the Centre hospitalier universitaire (CHU) de Québec. References came from a qualified neurologist (RL), experienced in the management of neurodegenerative diseases. Clinical diagnosis for svPPA patients was made based on currently accepted criteria (Gorno-Tempini et al., 2011). AD patients were diagnosed according to the research criteria of the National Institute of Neurological and Communicative Disorders and Stroke–Alzheimer’s Disease and Related Disorders Association (McKhann et al., 2011) and the clinical criteria for dementia of the Alzheimer type (American Psychiatric Association, 2000). The exclusion criteria were the following: history of traumatic brain injury or psychiatric disorder, developmental learning disabilities, uncorrected hearing and vision problems, first spoken language other than French and left-handedness. This study was carried out in accordance with the recommendations of the research ethics committee of the *CHU de Québec* with written informed consent from all subjects. All subjects gave written informed consent in accordance with the Declaration of Helsinki. The protocol was approved by the research ethics committee of the CHU de Québec (Project #2015-1909).

**Table 1:** Demographic information and neuropsychological data for the healthy control participants (HC), patients with the semantic variant of primary progressive aphasia (svPPA), and control patients with Alzheimer’s disease (AD).

	HC (*n* = 11)	svPPA (*n* = 9)	AD (*n* = 12)	*p*-value	Group comparison
**Demographics**					
Age	65.66 (8.1)	65.19 (11.2)	71.00 (8.2)	0.26	HC = svPPA = AD
Sex (F/M)	4/7	2/7	5/7	N/A	N/A
Education (years)	16.45 (3.1)	16.11 (4.1)	16.25 (3.0)	0.97	HC = svPPA = AD
**Global cognitive status**					
MMSE	28.9 (0.7)	25.22 (2.1)	25.08 (2.8)	<0.001***	HC > svPPA = AD
**Language and semantic Memory**					
BNT (/60)	49.91 (4.4)	12.67 (8.7)	42.67 (10.6)	<0.001***	HC = AD > svPPA
Irregular word-picture matching (/60)	52.09 (3.14)	24.89 (12.13)	49.33 (3.94)	<0.001***	HC = AD > svPPA
PPTT (/52)	50.18 (1.5)	31.67 (12.4)	48.17 (2.4)	<0.001***	HC = AD > svPPA
Semantic fluency	26.27 (4.4)	9.11 (7.8)	14.17 (7.1)	<0.001***	HC > svPPA = AD
Orthographic fluency	27.00 (8.8)	13.22 (5.7)	20.42 (8.0)	0.002***	HC > svPPA
Free fluency	67.64 (17.3)	30.67 (12)	39.92 (15.0)	<0.001***	HC > svPPA = AD
**Episodic memory**					
RAVLT 1-5	52.91 (7.4)	29.5 (7.8)	28.33 (5.9)	<0.001***	HC > svPPA = AD
RAVLT (delayed free recall)	10.82 (2.7)	4.67 (2.7)	1.92 (2.9)	<0.001***	HC > svPPA = AD
Rey-Osterrieth (delayed recall; /36)	20.27 (4.6)	8.44 (5.2)	5.29 (4.8)	<0.001***	HC > svPPA = AD
**Visuospatial perception**					
Benton Lines (/30)	27.45 (2.2)	26.56 (2.4)	24.17 (6.8)	0.22	HC = svPPA = AD
Rey-Osterrieth (copy; /36)	32.27 (2.7)	29.67 (4.5)	27.50 (8.0)	0.15	HC = svPPA = AD
**Executive functions and working memory**					
Stroop WC (sec)	127.36 (32.6)	135.12 (35.8)	223.00 (115.06)	0.01*	HC = svPPA > AD
Digit span (forward)	10.91 (2.6)	9.11 (2.1)	9.75 (1.3)	0.15	HC = svPPA = AD
Digit span (backward)	7.27 (2.5)	5.44 (2)	6.00 (2.0)	0.17	HC = svPPA = AD

*^∗^*p* < 0.05, ^∗∗∗^*p* < 0.001.*

### Neuropsychological Assessment

All participants underwent an exhaustive battery of standard neuropsychological tests. The battery assessed general cognitive status (Mini-Mental State Examination, Folstein et al., 1975). Folstein et al. (1975), as well as a number of cognitive domains. These domains include language and semantic memory (Boston Naming Test, Kaplan et al., 1983; Pyramids and Palm Trees Test, Howard and Patterson, 1992; Free fluency, orthographic and semantic fluency, Joanette et al., 2004), verbal and non-verbal episodic memory (Rey Auditory Verbal Learning Test, Rey, 1964; Delayed recall of the Rey Complex Figure Test, Osterrieth, 1944; Meyers and Meyers, 1995), visuospatial perception (Benton Line Orientation test, Benton et al., 1983; Qualls et al., 2000; copy of the Rey Complex Figure Test, Osterrieth, 1944; Meyers and Meyers, 1995), executive functioning (Stroop-Victoria Test, Spreen and Strauss, 1998) and working memory (Forward and Backward Digit-span, Wechsler, 1997). The test results are presented in **Table 1**.

### Stimuli

The experimental reading task comprised 60 low-frequency exception words, 60 low-frequency regular words and 60 pseudowords. This task has been previously used in Wilson et al. (2012), Chapleau et al. (2015), and Boukadi et al. (2016). Means for the psycholinguistic characteristics of each word type are presented in **Table 2**. The full list of stimuli and their characteristics can be found in Boukadi et al. (2016). Exception words violated the grapheme-to-phoneme conversion rules of French (Ziegler et al., 2003; Mechelli et al., 2005). For example, the French word *orchidée* (*orchid*) is an exception word as it is pronounced /ɔ
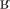
kide/, which does not follow the French rule according to which ‘ch’ is pronounced /∫/, as in *chocolat* (*chocolate*). Regularization errors could then be made with exception words if they were read in accordance with French rules (e.g., *orchidée* pronounced /ɔ
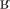
∫ ide/). On the contrary, regular words followed French rules (e.g., the French word *approche* (*approach*) is pronounced /ap
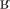
ɔ∫/). Pseudowords (e.g., *fuche*) were composed of legal (pronounceable) strings of letters that did not correspond to real words in French. Consequently, pseudowords had no semantic representations and had to be read on the basis of the grapheme-to-phoneme conversion rules of French. Lists of exception and regular words were matched for word frequency and imageability (*p*-values were non-significant). In addition, the three word lists were matched by initial phoneme, bigram frequency, length in letters, and orthographic neighborhood size (all *p*-values at least > 0.08). Psycholinguistic values were all taken from the French Lexical Database Lexique 3.01^1^ (New et al., 2004) except for bigram frequency values, which were taken from the WordGen programme^2^ (Duyck et al., 2004).

**Table 2:** Means and standard deviations (SD) for the psycholinguistic characteristics of each word type.

	Exception words		Regular words		Pseudowords	
Psycholinguistic characteristics	Mean	*SD*	Mean	*SD*	Mean	*SD*
Frequency	5.02	7.03	5.22	6.41	-	-
Imageability	4.55	1.62	4.65	1.59	-	-
SumTypeBiFreq	11223.65	6373.38	12941.12	5263.13	12465.65	7565.91
Length in letters	5.57	1.51	5.95	1.16	5.52	1.51
Length in syllables	1.63	0.69	1.83	0.64	-	-
Length in phonemes	4	1.55	4.68	1.07	4.5	1.6
Orthographic neighborhood	1.87	3.01	2.55	3.25	3.03	2.29
Phonological neighborhood	8.75	9.50	5.95	7.21	-	-
Homographs	1.18	0.43	1.27	0.66	-	-
Homophones	3.73	3.13	3.00	1.89	-	-

*Word written frequency is on one million tokens; Imageability is given as seven-point subjective ratings; Homographs: number of homograph words; Homophones: number of homophone words; SumTypeBiFreq, length in letters, syllables and phonemes, orthographic neighborhood, phonological neighborhood, homographs and homophones are reported as absolute values. SumTypeBiFreq, summed-type bigram frequency.*

### Procedure

We presented the stimuli on a PC using the DMDX software (Forster and Forster, 2003). Participants were asked to read aloud the pseudowords and words as accurately and as quickly as possible. The 60 pseudowords were presented in two separate blocks of 30 stimuli each. All the words (exception and regular words) were randomized and presented together in 4 blocks of 30 stimuli each (with 15 exception and 15 regular words each). We randomized the order of block presentation as well as the order of the stimuli within each block. Pseudowords were presented in two separate blocks of 30 items each. We presented pseudowords separately from words to encourage participants to read regular words using a whole-word strategy rather than a subword one. Indeed, list-context manipulation may favor either whole-word or subword reading strategies (Monsell et al., 1992; Lupker et al., 1997). Before reading pseudowords and words, participants were presented 10 practice stimuli. At the beginning of each trial, a blank screen was presented during 400 ms, followed by a fixation cross that appeared in the center of the screen for 400 ms. The target pseudoword or word then appeared in lowercase 15 pt Arial font for a maximum of 2,000 ms. Recorded vocal responses were then corrected and scored using the CheckVocal programme (Protopapas, 2007). This programme presents each recorded response audiovisually with sound, waveform and spectrogram, which allowed us to check the accuracy off-line and to retrigger vocal response onset when necessary.

Errors were classified as follows: (1) null responses; (2) syllabifications; (3) paralexias; (4) errors with complex OPM (for regular words and pseudowords only), (5) regularizations (for exception words only); and (6) lexicalisations (for pseudowords only). Null responses included no responses and incomplete responses. Syllabifications occurred when a word was separated into syllables. Paralexias included phonemic omissions, additions and substitutions [e.g., the word *cancre* (*dunce*) read /k
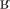
ãk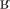/ instead of /kãk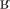/]. Errors with complex OPM occurred when the correspondences did not follow the contextual rules of French for the graphemes ‘s, g, c’ or the conversion rules for multiletter graphemes (Burani et al., 2006; Auclair-Ouellet et al., 2013). Indeed, in French, ‘s’ is pronounced as /z/ when it comes between two vowels. This means that the regular word *asile* (*asylum*) had to be pronounced /azil/. Thus, we classified the pronunciation /asil/ as an error regarding complex OPM. Also, in French, the graphemes ‘g’ and ‘c’ are pronounced /g/ and /k/, respectively, when preceding the vowels ‘a, o, u.’ However, when ‘g’ and ‘c’ precede the vowels ‘i, e, y’ they are pronounced /ʒ/ and /s/, respectively. OPM complexity in regular words was classified *post hoc*. We considered that a grapheme corresponded to a complex OPM when this grapheme was classified as complex and/or contextual according to the French classification of Pérez (2014). We also classified as complex OPM letters that must be silent at the end of words (e.g., the letter ‘t’ is silent in the word *ourlet* (*hem*), as it is for the vast majority of words that end by the letters ‘et’ in French). In French, a large number of letters are systematically silent in particular contexts (Perry et al., 2014), which means that the reader needs to consider the letter context to determine its correct pronunciation. Among the 60 regular words, 48 contained at least one complex OPM. A regularization error was made when a participant read an exception word according to the OPM (e.g., the letters ‘ch’ in the word *orchid* pronounced /∫/ as in the word *chocolat* rather than /k/) (Patterson and Hodges, 1992; Wilson et al., 2009, 2012). A lexicalisation occurred when a participant read a pseudoword as a real word, with formal similarity between them [e.g., the pseudoword *nercure* read as the word *mercure* (*mercury*); Dos Santos et al., 2013]. Of note, because pseudowords have no specific pronunciation, we accepted all plausible pronunciations that followed French rules. We used the French Lexical Database Lexique 3.01^3^ (New et al., 2004) to determine the pronunciations that were acceptable. We determined that plausible pronunciations that followed French rules were those in which grapheme pronunciations corresponded to at least 10% of the words having the same grapheme(s) in the same position in the Lexique.org database. For example, for the pseudoword *drin*, we accepted the pronunciation /d
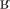

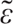
/ but we did not accept /d
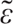
in/ considering that 97.2% of the French words ending with the graphemes ‘in’ are pronounced /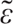/ as opposed to 2.8% of them that are pronounced /in/.

### Statistical Analyses

We used the linear mixed effects modelling approach, a type of analysis that controls for the crossed random effects of participants and items (Baayen et al., 2008), with word type (exception words vs. regular words vs. pseudowords) and group (controls vs. svPPA vs. AD) as fixed effects. Accuracy, reaction times, and number of regularizations and errors with complex OPM were entered in the models as dependent variables. Reaction time analyses were conducted on correct responses only. Simple effect analyses were conducted to study significant interactions. Statistical analyses were conducted with SPSS24. We considered results with a *p*-value < 0.05 significant.

### Voxel-Based Morphometry

#### Image Acquisition

All participants underwent a structural MRI scan. Imaging was performed on a 3T Philips Achieva TX scanner at IRM Québec-Mailloux in Québec City. High-resolution T1-weighted structural images were acquired with a volumetric magnetization prepared rapid gradient echo (MP-RAGE) sequence (repetition time = 8.2 ms, echo time = 3.7 ms, field of view = 250 mm, flip angle = 8°, 256 × 256 matrix, 180 slices/volume, slice thickness = 1mm, no gap).

#### Image Preprocessing

We performed VBM analyses using the Statistical non-Parametric Mapping toolbox^4^ for SPM12 (Welcome Department of Imaging Neuroscience, London, UK^5^). The structural images were first segmented into GM, white matter and cerebrospinal fluid. We further created a specific template for this study using the diffeomorphic anatomical registration through an exponentiated Lie algebra algorithm (DARTEL; Ashburner, 2007). All grey matter images were warped to the custom template and then spatially normalised into Montreal Neurological Institute (MNI) space. To compensate for the effect of spatial normalisation, the spatially normalized grey matter was adjusted by multiplying its relative volume before warping. The modulated images were then smoothed with a Gaussian kernel of 8 mm.

#### Statistical Analyses

For each of the two error types of interest (regularization errors for exception words and errors with complex OPM during regular word reading), we entered the number of errors of each participant as a covariate of interest in multiple regression statistical models with age, gender and group (svPPA, AD and healthy control participants) included as nuisance covariates. We followed the same procedure for the accuracy in pseudoword reading as a control condition, since we do not expect to find a significant correlation between reading performance of this word type and GM volume in the left ATL. We entered smoothed GM images of all participants as a single group in these statistical models. We set specific contrasts to identify the brain regions whose GM volume correlated with the number of regularizations and errors with complex OPM and accuracy in pseudoword reading. We tested the correlations with a [-1] *t*-contrast for number of errors and a [1] *t*-contrast for pseudoword reading accuracy, postulating that these variables of interest would correlate in a negative and positive way, respectively, with GM volume. Whole brain analyses were conducted using a statistical threshold of *p* < 0.001 uncorrected for multiple comparisons.

## Results

### Reading Task

#### Accuracy

**FIGURE 1:**
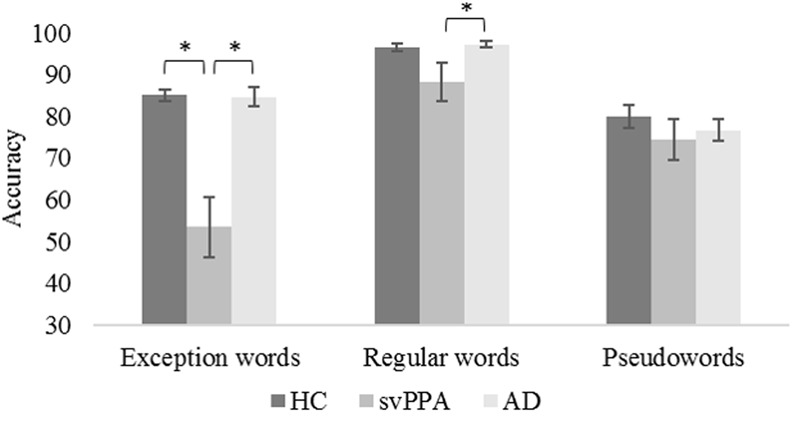
**Mean accuracies and standard errors of the mean as a function of group and word type.** HC, healthy control group; svPPA, semantic variant of primary progressive aphasia; AD, Alzheimer’s disease control group. ^∗^*p* < 0.05.

**Table 3** shows the mixed model analysis estimates and tests of fixed and simple effects by accuracy. The group significantly affected accuracy (*p* < 0.001). We illustrated mean correct response rates and standard errors of the mean for each group in **Figure 1**. The interaction Group by Word type was also significant. The analysis of the interaction indicated significant main effects of group for exception words (*p* < 0.001) and regular words (*p* < 0.05). No group effect was observed for pseudowords (*p* = 0.19). Critically, for exception words, simple effects analyses revealed that the mean correct response rate was lower for the svPPA group (*M* = 53.4, *SD* = 20.4) than for the healthy control (*M* = 85.1, *SD* = 4.6) and AD control groups (*M* = 84.7, *SD* = 7.3). For regular words, the mean correct response rate was significantly lower for the svPPA group (*M* = 88.2, *SD* = 13.3) as compared to the AD control group (*M* = 97.3, *SD* = 2.8). The performance difference between the svPPA group and the healthy control group (*M* = 96.5, *SD* = 3.2) was marginally significant.

**Table 3:** Mixed model analyses estimates and tests of fixed and simple effects by accuracy.

Word type	Parameter	*F*	Numerator *df*	Denominator *df*	*p*-value
All 3 stimuli types	Intercept	1766.96	1	65.53	<0.001***
	Group	11.03	2	28.22	<0.001***
	Word type	186.66	2	5055.80	<0.001***
	Group X Word type	5052.71	4	5052.70	<0.001***
Exception words	Intercept	549.15	1	74.79	<0.001***
	Group	23.28	2	28.17	<0.001***
	svPPA vs. HC	27.43	1	17.48	<0.001***
	svPPA vs. AD	24.68	1	18.82	<0.001***
	HC vs. AD	0.31	1	21.21	0.58
Regular words	Intercept	4896.83	1	30.82	<0.001***
	Group	4.56	2	27.32	0.02*
	svPPA vs. HC	4.15	1	17.09	0.06
	svPPA vs. AD	5.50	1	18.15	0.03*
	HC vs. AD	0.40	1	21.14	0.53
Pseudowords	Intercept	438.44	1	70.22	<0.001***
	Group	1.79	2	28.12	0.19

*svPPA, semantic variant of primary progressive aphasia; HC, healthy control group; AD, Alzheimer’s disease control group. ^∗^*p* < 0.05, ^∗∗∗^*p* < 0.001.*

#### Reaction Times

**FIGURE 2:**
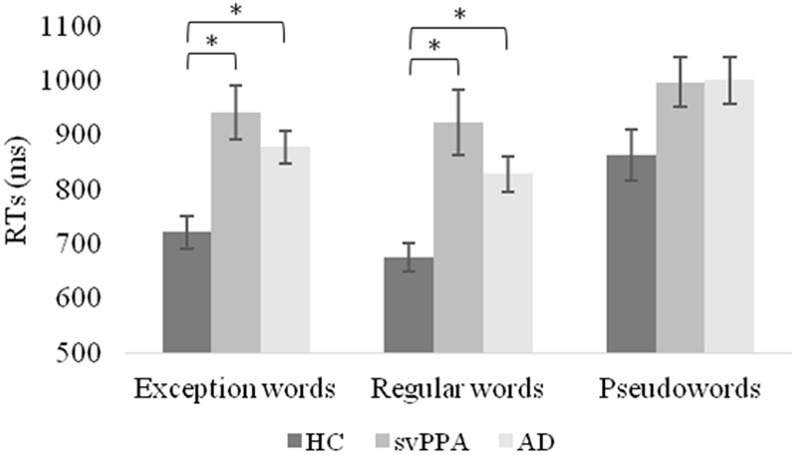
**Mean reaction times and standard errors of the mean as a function of group and word type.** HC, healthy control group; svPPA, semantic variant of primary progressive aphasia; AD, Alzheimer’s disease control group; ms, milliseconds. ^∗^*p* < 0.05.

**Table 4** shows the mixed model analysis estimates and tests of fixed and simple effects by reaction times. The group significantly affected reaction times (*p* < 0.001). We reported mean reaction times and standard errors of the mean for each group in **Figure 2**. The interaction Group by Word type was also significant. The analysis of the interaction indicated significant main effects of group for exception words (*p* < 0.001) and regular words (*p* < 0.001), but not pseudowords (*p* = 0.05). For exception words, the svPPA group was significantly slower (*M* = 941.72, *SD* = 142.59) than the healthy control group (*M* = 721.33, *SD* = 92.40). Reaction times of the svPPA group in exception word reading did not differ from those of the AD control group (*M* = 877.73, *SD* = 98.22). For regular words, participants of the svPPA group were also slower (*M* = 923.40, *SD* = 168.95) than those of the healthy control group (*M* = 675.14, *SD* = 82.07). Reaction times of the svPPA patients did not differ from those of the AD control group (*M* = 828.40, *SD* = 106.05).

**Table 4:** Mixed model analyses estimates and tests of fixed and simple effects by reaction times.

Word type	Parameter	*F*	Numerator *df*	Denominator *df*	*p*-value
All 3 types of stimuli	Intercept	1622.19	1	34.72	<0.001***
	Group	8.56	2	28.85	0.001***
	Word type	208.26	2	4218.62	<0.001***
	Group X Word type	4187.32	4	4187.32	<0.001***
Exception words	Intercept	1330.88	1	46.18	<0.001***
	Group	11.23	2	28.10	<0.001***
	svPPA vs. HC	19.12	1	17.50	<0.001***
	svPPA vs. AD	1.64	1	18.39	0.22
	HC vs. AD	14.74	1	20.56	0.001***
Regular words	Intercept	1214.76	1	37.96	<0.001***
	Group	11.13	2	28.55	<0.001***
	svPPA vs. HC	19.15	1	17.70	<0.001***
	svPPA vs. AD	2.49	1	18.68	0.13
	HC vs. AD	14.61	1	20.81	0.001***
Pseudowords	Intercept	1025.61	1	48.12	<0.001***
	Group	3.24	2	28.73	0.05

*svPPA, semantic variant of primary progressive aphasia; HC, healthy control group; AD, Alzheimer’s disease control group. ^∗∗∗^*p* < 0.001.*

#### Type of Errors

A qualitative analysis revealed that for exception word reading, regularizations were the most frequent errors. **Table 5** shows the mixed model analysis estimates and tests of fixed and simple effects by error type. The group significantly affected the number of regularizations (*p* < 0.001). Simple effect analyses indicated that svPPA patients made a greater number of regularizations (*M* = 19, *SD* = 8.2) as compared to healthy control participants (*M* = 5.9, *SD* = 1.8) and AD control patients (*M* = 6.6, *SD* = 3.6). Regarding regular word and pseudoword reading, errors with complex OPM were the most prevalent errors. For regular word reading, the group significantly affected the number of complex OPM (*p* < 0.01). The svPPA group made a greater number of errors related to complex OPM (*M* = 2, *SD* = 2.5) as compared to the healthy control (*M* = 0.2, *SD* = 0.4) and AD control groups (*M* = 0.2, *SD* = 0.6). For pseudoword reading, there was no group effect for the number of complex OPM (*p* = 0.33).

**Table 5:** Mixed model analyses estimates and tests of fixed and simple effects by error type.

Error type	Parameter	*F*	Numerator *df*	Denominator *df*	*p*-value
Regularizations	Intercept	47.22	1	75.45	<0.001***
*(Exception words)*	Group	21.50	2	29.00	<0.001***
	svPPA vs. HC	26.93	1	18.00	<0.001***
	svPPA vs. AD	22.26	1	19.00	<0.001***
	HC vs. AD	0.31	1	21.00	0.58
Complex OPM	Intercept	7.82	1	40.86	0.008***
*(Regular words)*	Group	5.66	2	29.00	0.008***
	svPPA vs. HC	5.70	1	18.00	0.03*
	svPPA vs. AD	6.12	1	19.00	0.02*
	HC vs. AD	0.005	1	21.00	0.94
Complex OPM	Intercept	22.63	1	74.09	<0.001***
*(Pseudowords)*	Group	1.16	2	29.00	0.33

*svPPA, semantic variant of primary progressive aphasia; HC, healthy control group; AD, Alzheimer’s disease control group. ^∗^*p* < 0.05, ^∗∗∗^*p* < 0.001.*

### Voxel-Based Morphometry Results

**FIGURE 3:**
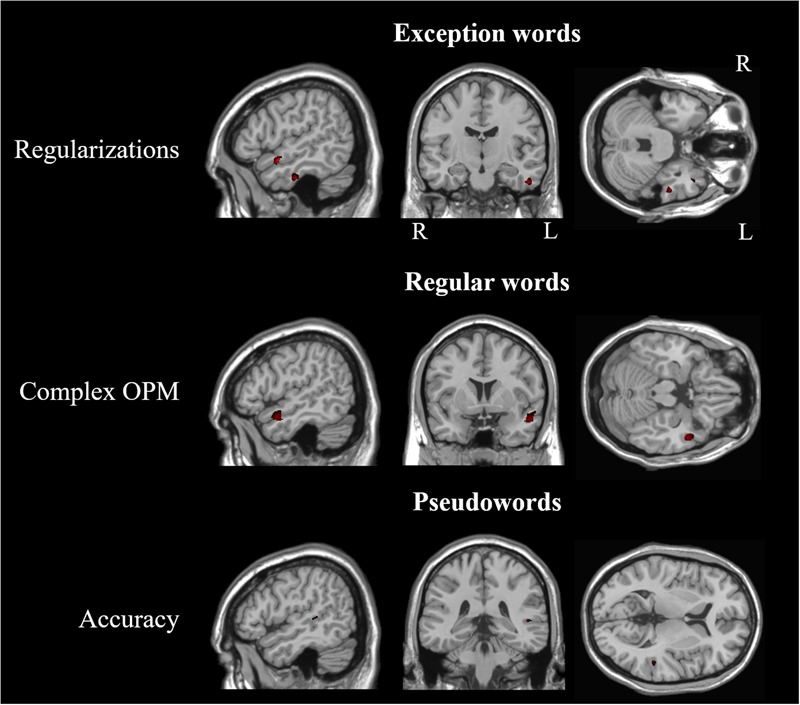
**Statistical maps depicting brain regions in which gray matter volume correlated with the number of errors during whole-word reading, and with accuracy in pseudoword reading.** L, Left; R, Right. *Z*-statistic maps (*p* ≤ 0.001 uncorrected) are displayed on three intersecting (sagittal, coronal, transaxial) slices.

Significant correlations between GM volume and number of errors in our whole brain analyses are reported in **Table 6** and **Figure 3**. We found significant correlations between the number of regularizations and GM volume of lateral and ventral portions of the left ATL (middle and inferior temporal gyri) and left insula. Regarding the number of complex OPM, we found significant correlations with GM volume of the lateral portion of the left ATL (middle temporal gyrus) and left insula. Brain regions in which GM volume correlated significantly with pseudoword reading accuracy in our whole brain analyses are reported in **Table 7**. Significant correlations were found in the following left hemisphere regions: posterior middle temporal gyrus, angular gyrus and middle frontal gyrus. In order to study to what extent the whole-brain correlations were driven or not by structural differences in the group of svPPA, known to have ATL atrophy, we conducted *post hoc* analyses for each of the two error types of interest with the diagnostic of svPPA versus healthy control and AD participants together as nuisance covariate. The analysis conducted with the number of regularizations revealed significant correlations with GM volume in the following regions of the left hemisphere: insula, posterior superior temporal gyrus and superior occipital gyrus. The analysis performed with the number of complex OPM showed significant correlations in the right superior parietal gyrus and the left cuneus. No correlation was found in the left ATL for both analyses with the diagnostic of svPPA as nuisance covariate.

**Table 6:** Montreal Neurological Institute peak coordinates for significant VBM correlations with number of regularizations, and number of errors with complex OPM during regular word reading.

				MNI coordinates				
Error type	Hemisphere	Location	BA	*X*	*Y*	*Z*	*T*-value	*P* uncorr.
Regularizations	Left	Anterior MTG	21	-51	2	-17	7.22	0.0002
		Insula	48	-41	14	-3	6.64	0.0002
		Anterior ITG	20	-53	-15	-32	6.56	0.0002
Complex OPM *(Regular words)*	Left	Anterior MTG	21	-53	5	-20	4.07	0.0008
		Insula	48	-42	12	-5	3.71	0.0008

*ITG, Inferior temporal gyrus; MTG, Middle temporal gyrus.*

**Table 7:** Montreal Neurological Institute peak coordinates for significant VBM correlations with pseudoword reading accuracy.

			MNI coordinates				
Hemisphere	Location	BA	*X*	*Y*	*Z*	*T*-value	*P* uncorr.
Left	Posterior MTG	21	-48	-36	5	5.06	0.0002
	Angular gyrus	39	-50	-69	38	4.36	0.0004
	Middle frontal gyrus	9	-26	29	45	4.34	0.0004

*MTG, Middle temporal gyrus.*

## Discussion

The ventral and lateral ATLs have been recently associated with semantic processing. Nevertheless, their role during exception and regular word reading (i.e., words with semantic representations) required further investigation. The first goal of this study was to characterize the reading abilities in svPPA patients. In accordance with our hypotheses, svPPA patients were less accurate and committed more regularization errors as compared to the healthy and AD control groups for exception word reading. This finding is consistent with those of previous studies (Wilson et al., 2012; Woollams et al., 2014; Boukadi et al., 2016) and suggests a profile of surface dyslexia for svPPA patients. Regarding regular words, svPPA patients had a lower accuracy as compared to AD patients only. It is possible that the performance difference between svPPA and healthy controls would have been significant with larger sample sizes, as the comparison was marginally significant. Nevertheless, svPPA patients produced a greater number of errors with complex OPM, as compared to the control groups of healthy participants and AD patients. Their semantic impairment probably accounted for this result. To test this hypothesis, and following Woollams et al. (2007), we performed Pearson’s correlations between a composite semantic score and the number of regularizations and complex OPM in svPPA participants. The composite semantic score included performance in picture naming (Kaplan et al., 1983) and exception word-picture matching (Chapleau et al., 2015) tasks. The latter task tested comprehension of the exception words presented in the experimental reading task. We found significant negative correlations between the semantic composite score and both the number of regularizations (*r* = -0.70, *p* < 0.05) and the number of complex OPM errors (*r* = -0.68, *p* < 0.05). This means that the larger the semantic impairment svPPA participants had, the more regularization and complex OPM errors they committed. This provides support to our hypothesis that it is semantic knowledge impairment that could have accounted for the error performance of svPPA patients. In fact, semantic knowledge can support both exception and regular word reading (Plaut et al., 1996; Strain and Herdman, 1999; Davies et al., 2013; Ricketts et al., 2016). Thus, although regular words with complex OPM can be correctly read through sublexical processes, their reading might be facilitated by the use of the whole-word reading network. Interestingly, Auclair-Ouellet et al. (2016) found that the semantic impairment in svPPA patients affected morphological processes for both exception and regular verbs. Thus, the degradation of semantic knowledge conveyed by units such as morphemes or graphemes would not solely impact exception word processing but also regular words with complex OPM. In the present study, we argue that svPPA patients, due to their semantic impairment, could not rely on an intact whole-word reading network to read aloud regular words with complex OPM properly, as compared to healthy participants. The svPPA group also had longer reading reaction times for exception and regular words, but not pseudowords, as compared to the healthy control group. This result supports the idea of an impairment of whole-word reading in svPPA patients.

Regarding our AD control patients, they had intact reading accuracy as expected. However, they had longer reading latencies compared to the healthy control group, as previously observed (Passafiume et al., 2000; Gold et al., 2005). This pattern was found for exception and regular words but not for pseudowords. Thus, this result could reflect a specific slowing of whole-word reading processes alone. This idea is in line with the findings of Passafiume et al. (2000). Their study suggests that semantic impairment affects word reading latencies in AD patients. Indeed, they found a selective increase of reading latencies for words that were mismatched or misnamed (i.e., for words with impaired semantics). Reaction times have been reported to be a more sensitive measure than accuracy to study reading abilities in AD patients (Pirozzolo et al., 1988). Thus, reading latencies are a good index of the efficiency of whole-word processes and slower reading latencies could precede the subsequent reduced word reading accuracy found in the later stages of the disease, especially for exception words (Patterson et al., 1994). In light of this, we would predict that the early onset AD patients in our control group would also develop reading accuracy difficulties with disease progression. Early-stage AD patients typically show atrophy in the temporal lobes, but in regions other than the ATLs. Normally, they show temporoparietal GM atrophy as compared to healthy controls (Whitwell et al., 2011). These posterior regions of the left middle temporal gyrus, along with the angular gyrus, have been associated with whole-word reading processes (Taylor et al., 2013). Thus, atrophy in these regions in AD patients might account for the slowing in reaction times for exception and regular word reading.

The second goal of this study was to investigate whether GM volume of the ATLs was associated with the number of errors in reading exception and regular words. In accordance with our hypotheses, the volume of the left ATL was associated with the number of exception word regularizations and the number of regular word errors with complex OPM. Specifically, we found that lateral and ventral parts of the left ATL correlated with the number or regularizations. This result suggests that the integrity of the left ATL is critical to the reading of unpredictable OPM. The findings of Shimotake et al. (2015), together with our results, suggest that the left ventral ATL plays a key role in exception word reading in both ideographic (Japanese Kanji) and alphabetical scripts (French).

Regarding the number of complex OPM errors, we found an association between this type of error during regular word reading and the lateral region of the left ATL. Thus, the left lateral ATL might be involved in the processing of both atypical spelling-to-sound correspondences and complex OPM. The left lateral ATL receives connections from a ventral pathway and a dorsal pathway, which have been associated with semantic and phonological processing, respectively (Vigneau et al., 2006; Hickok and Poeppel, 2007; Richardson et al., 2011; Yvert et al., 2012). Incidentally, the left lateral ATL has been identified as a high-order heteromodal cortex (Spitsyna et al., 2006), involved in combinatorial processes, along with posterior regions of the temporal cortex (Hickok and Poeppel, 2007; Hickok, 2012). Specifically, the connections between the posterior superior temporal sulcus and the anterior superior temporal sulcus could be involved in the integration of phonology and semantics (Richardson et al., 2011), that might apply to the computation of complex OPM present in regular words. Nevertheless, Hoffman et al. (2015) did not observe a pattern of increasing activity within the left lateral ATL for regular words. This discrepancy might be due to cross-language differences. The French script comprises a greater percentage of complex rules (i.e., context sensitive correspondences and multiletter graphemes), as compared to English (Schmalz et al., 2015). Thus, our regular words might have been more complex than those used in Hoffman et al. (2015), and might have been associated with the left lateral ATL to a greater extent than in their study. Our results suggest that the integrity of the left lateral ATL might be necessary to determine whether a pronunciation obtained by OPM corresponds to the whole-word representation of a familiar word. Such an ability to integrate phonological and semantic information would be essential to read unpredictable OPM in exception words accurately, but also regular words with complex OPM. Other studies found that the lateral ATLs were involved in processing familiar concepts, either presented in visual or auditory modality (Reilly et al., 2016), as well as in processing of both words and pictures (Visser et al., 2012; Chedid et al., 2016). Therefore, it is likely that the left lateral ATL plays a role in general semantic integration that is not limited to word reading.

Although we did not find a correlation between the ventral ATL and the number of complex OPM errors, the present results are not an argument against the involvement of the left ventral ATL in regular word processing, as previously found (Taylor et al., 2013; Hoffman et al., 2015). Our results suggest that at least the integrity of the left lateral ATL is critical for complex OPM reading. As regular words with complex OPM can be read through both sublexical and whole-word reading networks, reading regular words with complex OPM might be preserved despite a volume loss of the left ventral ATL. It is important to note that there are important methodological differences between our study and previous ones. In our study, we used the VBM technique and included neurological patients as well as healthy participants whereas other studies (i.e., Taylor et al., 2013; Hoffman et al., 2015) used fMRI and PET and included healthy participants only. This may at least partially explain the pattern of correlation with the left lateral ATL found in the present study.

Regarding theoretical models of reading, our findings are in accordance with the involvement of semantics for whole-word reading, as proposed by the PDP model (Seidenberg and McClelland, 1989; Plaut et al., 1996; Harm and Seidenberg, 2004). Indeed, our results show that GM volume in the ATLs, involved in semantics (Patterson et al., 2007; Price, 2010; Visser et al., 2010), is associated with the number of regularization errors. Moreover, GM volume in the lateral portion of the left ATL was associated with complex OPM errors in regular word reading. However, our study was not specifically designed to test the hypotheses of reading models and further explicitly designed studies are needed in order to shed light on this matter.

Among the limitations of our study we have to mention that the VBM results are non-corrected for multiple comparisons. Thus, our VBM results should be taken with extreme caution. Moreover, we discussed mainly the involvement of the ventral and lateral parts of the left ATL. However, Ding et al. (2009) identified at least seven regions in the human temporal polar cortex by analyzing different neuroanatomical markers. They also found subtle architectonic differences between the inferior temporal gyrus and the middle temporal gyrus, corresponding to areas 20 and 21 of Brodmann (1909). Finally, we acknowledge the limitations of the VBM technique itself to study the association between behavioral performance and GM volume in clinical populations. Indeed, *post hoc* VBM analyses with the diagnostic of svPPA included as nuisance covariate indicated that the pattern of association between errors and ATL regions was driven by the svPPA patients. Thus, further neuroimaging studies are needed to explore the involvement of the lateral and ventral ATLs for reading both unpredictable words and words with complex OPM in healthy participants. We also stress the fact that, consistent with the predictions of theoretical models based on behavioral and neuroimaging sources of evidence, only regular and exception words (that is to say, words that have semantic representations) were associated with the ventral and lateral ATLs and pseudowords, that do not have any semantic representation, were not associated with this brain region. In sum, the results of our study suggest that the left ATL might play a role in the reading of unpredictable OPM in exception words, in accordance with its role in semantic processing. Our findings also support the idea of the role of the left ATL, and especially its lateral part, in combinatorial processes including the integration of semantic and phonological information during both unpredictable and predictable OPM reading.

## Author Contributions

MJ analyzed the data, performed the statistical analysis, and drafted the manuscript. MW and SB designed the study and supervised data acquisition. RL referred and characterized the svPPA and AD patients. MM and MB collected the data. SB and MM also helped with the VBM analysis. IR, JM, SJ, and SF contributed to data interpretation. All authors revised the manuscript and approved the final version.

## Conflict of Interest Statement

The authors declare that the research was conducted in the absence of any commercial or financial relationships that could be construed as a potential conflict of interest.

## Footnotes

1 http://www.lexique.org/

2 http://expsy.ugent.be/wordgen.htm

3 http://www.lexique.org/

4 http://warwick.ac.uk/snpm

5 www.fil.ion.ucl.ac.uk/spm
